# Rational modification of substrate binding site by structure-based engineering of a cellobiose 2-epimerase in *Caldicellulosiruptor saccharolyticus*

**DOI:** 10.1186/s12934-017-0841-3

**Published:** 2017-12-12

**Authors:** Ah-Reum Park, Jin-Sook Kim, Seung-Won Jang, Young-Gyun Park, Bong-Seong Koo, Hyeon-Cheol Lee

**Affiliations:** ForBioKorea Co., Ltd., Gasan digital 2-ro, Geumcheon-gu, Seoul, Republic of Korea

**Keywords:** Lactulose, Cellobiose 2-epimerase, Lactose, Structure analysis, Protein engineering

## Abstract

**Background:**

Lactulose, a synthetic disaccharide, has received increasing interest due to its role as a prebiotic, specifically proliferating *Bifidobacilli* and *Lactobacilli* and enhancing absorption of calcium and magnesium. The use of cellobiose 2-epimerase (CE) is considered an interesting alternative for industrial production of lactulose. CE reversibly converts d-glucose residues into d-mannose residues at the reducing end of unmodified β-1,4-linked oligosaccharides, including β-1,4-mannobiose, cellobiose, and lactose. Recently, a few CE 3D structure were reported, revealing mechanistic details. Using this information, we redesigned the substrate binding site of CE to extend its activity from epimerization to isomerization.

**Results:**

Using superimposition with 3 known CE structure models, we identified 2 residues (Tyr114, Asn184) that appeared to play an important role in binding epilactose. We modified these residues, which interact with C2 of the mannose moiety, to prevent epimerization to epilactose. We found a Y114E mutation led to increased release of a by-product, lactulose, at 65 °C, while its activity was low at 37 °C. Notably, this phenomenon was observed only at high temperature and more reliably when the substrate was increased. Using Y114E, isomerization of lactose to lactulose was investigated under optimized conditions, resulting in 86.9 g/l of lactulose and 4.6 g/l of epilactose for 2 h when 200 g/l of lactose was used.

**Conclusion:**

These results showed that the Y114E mutation increased isomerization of lactose, while decreasing the epimerization of lactose. Thus, a subtle modification of the active site pocket could extend its native activity from epimerization to isomerization without significantly impairing substrate binding. While additional studies are required to scale this to an industrial process, we demonstrated the potential of engineering this enzyme based on structural analysis.

**Electronic supplementary material:**

The online version of this article (10.1186/s12934-017-0841-3) contains supplementary material, which is available to authorized users.

## Background

Recently, multiple cellobiose 2-epimerases (CE: EC 5.1.3.11) were reported for industrial process applications using lactose [1–4]. Specifically, CE converts lactose into the rare sugars, lactulose and epilactose (4-*O*-β-d-galactosyl-d-mannose), which are useful for medical applications as prebiotic agents [5–7]. Lactulose is a synthetic ketose disaccharide that is widely applied in the pharmaceutical and nutraceutical fields, due to its beneficial effects. Lactulose can be used as a gentle laxative for the treatment of constipation and hyper-ammonemia by stimulating growth of beneficial intestinal bifidobacteria but not inducing the proliferation of harmful bacteria belonging to classes Clostridia or Bacteroidetes [5, 7–9].

While there are current efforts to produce lactulose via enzymatic isomerization using CE [3, 4, 9–11], drawbacks exist, such as poor substrate solubility and low conversion yield, which need to be improved for industrial applications. It is well known that the substrate (lactose) has relatively low solubility (approx. 225 g/l at 37 °C) and most CEs partially change their isomerization/epimerization ratio at high temperature due to accelerating reaction velocity. Recently, CE from *Caldicellulosiruptor saccharolyticus* (CsCE) was reported as a useful enzyme having higher thermostability than other CEs [3, 12, 13]. Using this CsCE, we screened error-prone PCR-generated enzyme variants to identify those with increased thermostability compared to wild type CsCE [14]. Using a thermostable enzyme, the accelerated reaction at high temperature could have great advantages in producing lactulose, as lactulose is a byproduct produced during epimerization from lactose to epilactose, depending on equilibrium concentrations of lactose and epilactose [13, 15, 16].

CE was first identified in the ruminal anaerobic bacterium, *Ruminococcus albus* [17, 18], which catalyzes conversion of the glucose residue into a mannose residue or a fructose residue at the reducing end of β-1,4-linked oligosaccharides, including β-1,4-mannobiose, cellobiose, and lactose. Epimerases acting on carbohydrates and their derivatives can be divided into 25 groups (EC 5.1.3.) depending on their catalytic reactions and substrate specificities [19, 20]. While most epimerases, converting the configuration of non-anomeric hydroxyl groups, act on modified active substrates harboring phosphate groups or nucleotide diphosphate groups, CE catalyzes the epimerization of unmodified sugars at the C2 position. The crystal structures of CE from *R. albus* (RaCE [19]) and *Rhodothermus marinus* (RmCE [20]) were released recently. The crystal structures confirmed the structure proposed previously by Ito et al. [18, 21]. The basic conformation of CE consists of 12 α-helices arranged in an (α/α)_6_ barrel structure (Additional file 1: Figure S1). The handful of known CE sources (e.g. RaCE and RmCE) follow a similar epimerization mechanism and their structures adopt an (α/α)_6_ barrel structure similar to the catalytic domains of *N*-acetylglucosamine 2-epimerase (AGE) and aldose–ketose isomerase (YihS) [20]. CsCE is also predicted to follow the same epimerization mechanism, because it has an (α/α)_6_ barrel structure similar to the catalytic domains of other CEs and belongs to the AGE superfamily along with AGE and YihS.

In CEs, the active site, with its triple histidine center, is located inside the barrel [19]. Two essential histidine residues, corresponding to the general acid/base catalysts of AGEs, and a third histidine residue located at the bottom of the substrate binding site are required for catalysis. The two essential histidine residues corresponding to CsCE-His247 and CsCE-His377 are completely conserved in CE enzymes, supporting the importance of these residues [19, 20]. In contrast, the function of the third histidine in CE (RaCE-His184 and CsCE-His188), conserved in known CEs and YihS, was revealed recently [19, 20]. Many reports proposed that the third histidine residue and its neighbors affect deprotonation of the C2 atom by interacting with the O2 atom of reducing end sugars for formation of the *cis*-enediol intermediate [19–22]. Insight from these structural studies and proposed catalytic mechanism inspired us to improve the biological activity for production of lactulose. In an effort to find residues of CsCE that might improve biological activity, we turned our attention to neighboring residues, such as Tyr114 and Asn184, excluding His residues which play a role as a general acid/base important in stabilizing product binding.

Here, in order to enhance lactulose production from a high concentration of lactose at high temperature, we rationally engineered the binding site of a thermostable CsCE, which was identified in a previous screen, based on its structure [14]. Our hypothesis was that modification of residues involved in interacting with the mannose moiety of epilactose could change epimerization activity to isomerization activity toward lactulose production by inhibiting the conversion from the *cis*-enediol intermediate to epilactose. In other words, since lactulose is an isomerizing byproduct formed in the reaction epimerizing lactose to epilactose, we hypothesized that an isomerization from lactose to lactulose could be induced by increasing the reaction rate with higher temperature, while delaying the conversion of the *cis*-enediol intermediate into epilactose. To test this notion, the three-dimensional structure of CsCE was superimposed on the crystal structures of the CEs from *R. albus* (RaCE) and *R. marinus* (RmCE) with epilactose and lactulose analogues [19, 20], yielding targeted residues for site-directed mutagenesis to create mutant enzymes with modified substrate binding residues. Comparison of product yields with the mutant enzymes supported our hypothesis. This structure-based approach for converting enzyme properties from epimerization to isomerization is one example of the utility of protein engineering approaches to satisfy industrial needs for enzymatic process, especially using those involving isomerizing and epimerizing enzymes.

## Methods

### Materials

The restriction endonucleases *Nhe*I and *Eco*RI, T4 DNA ligase, and EX taq polymerase were obtained from Takara (Kyoto, Japan). The expression vector pET28a (+) was obtained from Novagen (Darmstadt, Germany). Plasmid DNA was purified by using DNA mini-preparation kits (Qiagen, Hilden, Germany). *Escherichia coli* BL21 (DE3) (New England Biolabs, Hertfordshire, UK) was used for protein expression. *C. saccharolyticus* DSM 8903 cell was purchased from DSMZ (Braunschweig, Germany). Standard sugars (lactose, epilactose, and lactulose) for high-performance liquid chromatography (HPLC) were purchased from Sigma-Aldrich (MO, USA).

### Gene cloning and expression of cellobiose 2-epimerase

The genomic DNA from *C. saccharolyticus* was extracted using the genomic DNA extraction kit (Qiagen, Hilden, Germany). The gene (1173 bp) encoding putative *N*-acyl-d-glucosamine 2-epimerase (Genebank Accession No. ABP65941) was amplified by PCR using *C. saccharolyticus* genomic DNA as a template with the following primers. The sequences of forward and reverse primers were 5′-GCT AGC ATG GAT ATT ACA AGG TTT AAG GAA GAT TTA AAA G-3′ and 5′-GAA TTC TTA GTC AAC CCT TTT TAT TAT CTC CAA ACA CAT TC-3′, respectively. The PCR product was cloned into pET28a (+) vector using *Nhe*I and *Eco*RI restriction sites. The gene was designed at the 5′ end, encoding a short histidine tag and thrombin site. About 50 ng of recombinant pET28a-CSCE plasmid DNA was transformed into *E. coli* BL21 (DE3) cells using the heat shock method, and transformants were selected on LB agar plate supplemented with kanamycin. DNA sequencing was conducted at the Macrogen facility (Seoul, Korea). The recombinant *E. coli* cells for protein expression were cultivated with shaking at 200 rpm, 37 °C with 30 μg/ml kanamycin until the OD_600_ reached 0.6. IPTG was added to the culture medium at 0.5 mM to induce enzyme expression and the culture was incubated at 16 °C for 16 h.

### Site-directed mutagenesis

Site-directed mutagenesis of CsCE was performed using the QuikChange site-directed mutagenesis kit (Agilent Technologies, CA, USA). Each desired amino acid replacement was generated by using two synthetic oligonucleotide primers. After 16 amplification cycles (95 °C for 30 s, 55 °C for 1 min, and 68 °C for 7 min) with *Pfu* Turbo DNA polymerase (Agilent Technologies, California, USA), the PCR products were treated with 1 U *Dpn*I, and then the nicked plasmid DNA with the desired mutation was transformed into competent cells of *E. coli* BL21 (DE3). The sequences of all variable mutants were confirmed by DNA sequencing.

### Purification of cellobiose 2-epimerase variants

The grown cells were harvested and disrupted using an ultra sonicator, on ice for 2 min in a 50 mM PIPES buffer (pH 7.5). The unbroken cells and cell debris were removed by centrifugation and *E. coli* proteins were removed by heat treatment as described previously [23]. The samples thus obtained were further purified by His-tagged affinity chromatography (HisTrap™, Amersham Biosciences). The fractions containing the target proteins were collected and dialyzed against 50 mM PIPES buffer (pH 7.5). The protein concentration was determined by the Lowry method with bovine serum albumin as a standard and homogeneity of the target proteins was assessed by SDS-PAGE.

### Lactose conversion assay

The composition of the reaction mixture in a total volume of 100 μl containing 50 mM PIPES buffer (pH 7.5), 20–200 g/l of lactose, and 50 μg of enzyme was routinely incubated at each tested temperature for defined lengths of time. The rates of formation of lactulose or epilactose from lactose were measured HPLC system with HILICpak VG-50 4E column (Shodex, Kawasaki, Japan). The reaction was stopped by the addition of HCl to the reaction mixture at a final concentration of 200 mM. The concentrations of lactulose, lactose, and epilactose in the resulting solutions were determined by Alliance 2690 HPLC system equipped with a Waters 2410 refractive index (RI) (Waters, Milford, USA) and a HILICpak VG-50 4E column. The column was eluted at 42 °C with 75% acetonitrile, 20% methanol, and 5% distilled water at a flow rate of 1 ml/min.

### Structure determination and refinement

Structural models of the CEs were obtained from the Protein Data Bank. The known crystal structures CEs (CsCE: PDB ID 4Z4L, RmCE: PDB ID 3WKF, 3WKG, 3WKH, 3WKI, and RaCE: PDB ID 3VW5) were aligned using tools in PyMOL software (https://www.pymol.org/). The structures of CsCE and ligand-bound CsCE were determined by the molecular replacement method with the program AutoMR in the Phenix program package [24] (Additional file 1: Table S1). The structure of apo-CsCE was solved using the structure of RmCE (PDB ID 3WKF) as a search model. Subsequently, ligand-bound CsCE was determined using the structure of apo-CsCE and ligand-bound RmCE (PDB ID 3WKH, 3WKG) as a search model. Rotation and translation functions were calculated using data of 30.0–1.67-Å resolution. Superimposition of ligand-bound RmCE and CsCE was also optimized by PyMOL by manual fitting. The root mean square deviation (RMSD) values of resulting structures were calculated for all atoms of the protein backbone for the ligand-bound CsCE structure, in order to characterize the amount by which a given selection of predicted molecules deviates from a defined position in space. NAMD 2.6 (http://www.ks.uiuc.edu/Research/namd/) input files for RMSD analysis were prepared with the VMD program (http://www.ks.uiuc.edu/Research/vmd/). The VMD program was also used for viewing the simulation results. The NAMD output files from minimization and equilibration of ligand-bound CsCE in a water sphere were used in order to calculate RMSD values and to analyze the extent of equilibration of the simulation. The refined model was simulated in an explicit water environment for 420 ps in an effort to refine the structure and to establish the stability of the model in general. The time history for the RMSD reached a maximum deviation of approximately 1.5 Å after 40 ps, which suggested that the system had converged to a stable structure, or at least a stable local minimum close to the starting structure.

## Results

### Selection of target residues for modifying binding properties

**Fig. 1:**
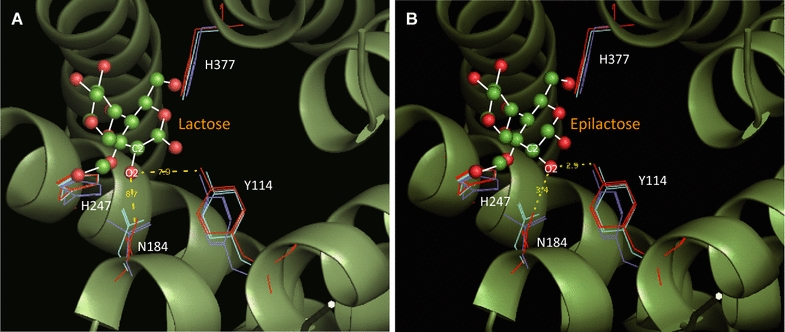
Superimposition of CsCE onto the RmCE-epilactose (lactose) complex based on the atoms of the 4 conserved active site residues. Based on this model of the binding site structure, the residues that are involved directly in the epimerization of the glucose moiety (His247 and His377) were observed to be highly conserved in the CE and AGE family. Additionally, Tyr114 and Asn184 were proposed to play a role in stabilizing the product and were observed to be closely associated with the stability of the enzyme-lactose/lactulose intermediate complex. **A** enzyme-lactose complex, **B** enzyme-epilactose complex

The sequence determined for CsCE obtained from *C. saccharolyticus* revealed that this CE gene was substantially different from that of other species and contained a 1173-bp ORF encoding 390 amino acids. Sequence alignment with known cellobiose epimerases revealed that all the enzymes have 12 α-helices, collectively termed the barrel (α_6_/α_6_) [20]. Their structures are quite similar to each other. The CsCE amino acid sequence shares 45% identity with RaCE and 38% with RmCE, but the structure of CsCE (4Z4L) shares conserved barrel (α_6_/α_6_) domains for cellobiose epimerization activities with all other CE family. Using the apo form of the CsCE structure, we used substrate binding simulations to model interactions with lactose, based on the structures of RmCE with ligands (3WKG) and RaCE (3VW5) (Fig. 1). As shown in Fig. 1, two essential histidine residues was located in the vicinity of the substrate binding site. Epimerization catalyzed by CE is likely to proceed with this pair of histidine residues, H377 and H247, which gives rise to a converting the configuration of C2 atom by abstracting and accepting a proton, according to the structural study for the epimerization mechanism of RmCE by Fujiwara et al. [20]. In addition to essential residues related to hydrolysis, there are residues thought to stabilize binding substrate or product. In Fig. 1B, the structure of the active site revealed that Y114 and N184 of CsCE are located at the interface of the mannose moiety of epilactose in the enzyme-product complex, and are thus easily able to interact with the hydroxyl group in the C2 position of mannose via H bonds. Y114 and N184 residues were observed to be involved in substrate binding but not directly in the lactose epimerization reaction. These residues (Y114 and N184) were considered to play a role in stabilizing the conversion of epilactose. Based on these observations, we hypothesized that the isomerizing byproduct (lactulose) can be produced at increased levels by endowing steric hindrance to the H-bonding of these residues with epilactose, a product of the epimerization reaction. If the enzyme binding sites for the reaction product (epilactose) were distorted by amino acid replacement without inhibiting substrate (lactose) binding, then the isomerizing byproduct, lactulose, can be released through *cis*-enediol intermediate, instead of completely proceeding through epimerization of lactose to epilactose.

### Mutagenesis of Y114 and N184 in CsCE

**Fig. 2:**
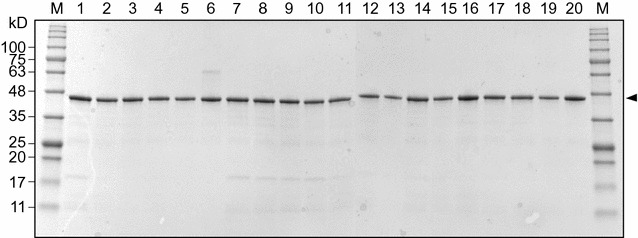
SDS-PAGE analysis of CsCE variants purified by Ni-affinity chromatography with heat-treated proteins

In order to test our hypothesis, the critical residues only involved in product stabilization, Y114 and N184, were targeted for site-directed mutagenesis studies. Y114 and N184 are well conserved in other enzymes in the cellobiose epimerase family. First, we prepared Y114X clones that covered all amino acid substitutions. All variants were expressed in soluble form and purified by heat treatment and were > 90% pure as judged by SDS-PAGE analysis (Fig. 2).

Our objective was to identify mutant enzymes that improved lactulose productivity by inhibiting epilactose conversion, while retaining affinity towards lactose. Thus, we investigated the isomerization activity from lactose to lactulose using the Y114X variants. Compared to the parental enzyme, most variants exhibited decreased isomerization activity from lactose to lactulose. The parental enzyme showed high epimerization activity toward epilactose at 37 °C (Table 1). Interestingly, even though most of Y114X mutants were found to lose activity entirely, the parental enzyme and the Y114E mutant enzyme showed increased isomerization activity from lactose to lactulose at 65 °C (Table 1). This was not surprising as recently CEs were used in the production of lactulose from lactose at higher temperature (60–70 °C) to enhance lactulose yield [3, 13]. Y114E, however, showed higher isomerization activity while producing less epilactose, compared to parental enzyme at 65 °C. As hypothesized, this result indicated that the formation of epimers through a *cis*-enediol intermediate to epilactose was inhibited by the adjacent mutated amino acid (Y114E), thereby promoting the release of lactulose as byproduct at 65 °C. At temperatures over 60 °C, compared to 37 °C, the isomeric byproduct lactulose could be released more readily due to the unstable substrate/product binding caused from an accelerated reaction rate. In addition to the Y114 mutation, we also investigated the conversion activity using an N184X library. All variants showed quite low conversion activity from lactose to lactulose as well as epilactose at both 37 and 65 °C (Additional file 1: Table S2). This may be due to more severe steric hindrance or charge repulsion compared to the Y114 position.

**Table 1:** Comparison of isomerase/epimerase activities on CsCE Y114X mutants at 37 and 65 °C

Amino acid substituted in Y114	37 °C		65 °C^a^	
	Isomerase activity (mM/min μg)	Epimerase activity (mM/min μg)	Isomerase activity (mM/min μg)	Epimerase activity (mM/min μg)
Y (parent)	15.6 ± 1.2	138 ± 2.3	187 ± 4.2	37.0 ± 3.6
V	9.74 ± 1.4	nd	29.2 ± 3.3	nd
N	5.84 ± 0.71	nd	9.73 ± 2.2	nd
S	1.95 ± 0.39	nd	17.5 ± 4.0	nd
A	9.74 ± 2.0	nd	25.3 ± 3.2	nd
K	9.74 ± 0.98	nd	15.6 ± 2.1	nd
G	3.90 ± 1.3	nd	15.6 ± 2.2	nd
D	11.7 ± 1.9	nd	17.5 ± 2.1	nd
C	5.84 ± 1.0	nd	15.6 ± 1.9	nd
L	nd	nd	29.2 ± 2.6	nd
E	17.5 ± 2.3	nd	208 ± 3.9	11.7 ± 2.2
P	1.95 ± 0.55	nd	17.5 ± 1.2	nd
I	7.79 ± 1.9	nd	17.5 ± 1.1	nd
W	nd	nd	11.7 ± 1.2	nd
T	3.90 ± 0.2	nd	7.81 ± 2.1	nd
R	nd	nd	13.6 ± 1.0	nd
F	1.95 ± 0.13	9.74 ± 2.1	48.7 ± 3.2	9.77 ± 3.2
M	3.90 ± 0.22	nd	17.5 ± 2.9	nd
Q	1.92 ± 0.08	nd	23.4 ± 3.4	nd
H	nd	nd	15.6 ± 2.8	nd

Isomerase and epimerase activities were measured by quantifying lactulose and epilactose which were converted from 20 g/l lactose for 30 min at testing temperature. Values are mean ± SD measured from three experimental replicates

nd, not detected

^a^Under this condition, lactulose formed by non-enzymatic isomerization was negligible

### Release of lactulose by incomplete epimerization

**Fig. 3:**
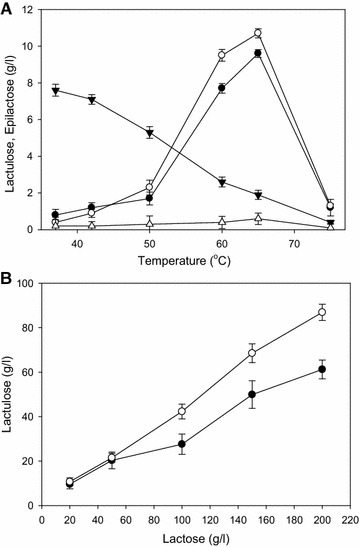
Effects of temperature and lactose concentration on activity using parental and Y114E enzymes. Lactulose (parental enzyme) (●), lactulose (Y114E enzyme) (○), epilactose (parental enzyme) (▼) and epilactose (Y114E enzyme) (△). **A** Effect of reaction temperature. Each 50 μg of enzyme was incubated with 20 g/l substrate solution at the range of 37–75 °C for 2 h. **B** Effect of lactose concentration. Each 50 μg of enzyme was incubated with 20–200 g/l substrate solution at 65 °C for 2 h

Substitution of Y114 with glutamate shifted product from epilactose to lactulose instead of completely abolishing the epimerization activity. The lactose conversion assay revealed that the rate of lactose consumption was considerably reduced at 37 °C but retained at 65 °C (Fig. 3A). As shown in Fig. 3A, the conversion of lactose into lactulose was temperature dependent and the activity of both enzymes was largely lost at 75 °C. The Y114E mutation significantly reduced epilactose production over the entire range, while the lactulose yield increased with increasing temperature. Interestingly, the isomerization reaction was observed only at high temperatures and more reliably as the substrate increased. Within the range tested for Y114E, lactulose production positively correlated with the lactulose concentration in the reaction mixture. This result indicated that the isomerization ratio of lactulose production was maintained with increasing lactose concentration, while the isomerization ratio of lactulose was decreased in the parental enzyme (Fig. 3B). Taken together, these results indicated that the increased reaction rate induced by high temperature may accelerate a release of the isomeric byproduct (lactulose), leading to maintenance of the isomerization ratio even at high concentrations of substrate (lactose). This finding is consistent with the structural analysis, as mentioned above.

### Production of lactulose using Y114E mutant enzyme

**Fig. 4:**
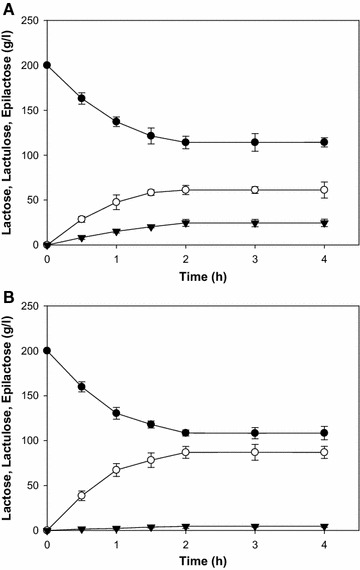
Conversion of lactose into lactulose in batch reaction using parental (**A**) and Y114E enzymes (**B**). Each enzyme was incubated with 200 g/l of substrate solution at 65 °C for 4 h. Lactose (●), lactulose (○), epilactose (▼)

To evaluate our Y114E enzyme, we tested lactulose production with the parental and Y114E enzymes under the condition described by Oh et al. [3, 13]. When 200 g/l of lactose was subjected to substrate under the reported conditions, Y114E produced 86.9 g/l of lactulose and 4.6 g/l of epilactose, while the parental enzyme produced 61.2 g/l of lactulose and 24.6 g/l of epilactose (Fig. 4). The isomerization of lactose no longer proceeded after 2 h, leading to an equilibrium state, whereby the ratio (lactose:lactulose:epilactose) in reaction mixtures containing the parental and Y114 enzyme were 57:31:12 and 53:45:2, respectively. These results showed that isomerization of lactose was increased by the Y114E mutation, while epimerization of lactose was decreased. Although RmCE have high activities of both epimerization and isomerization at mesophilic temperature, the relationship between its isomerization activity and the phyla of the originating organism was reported to be unclear [25]. However, at least in high substrate condition (over 100 g/l), most of CEs showed an increased isomerization activity than epimerization activity at high temperature. In this study, the subtle modification of the active pocket to inhibit stable binding to the end product (epilactose), could extend its activity from epimerization to isomerization without significantly impairing substrate binding at high temperature, thereby demonstrating the potential of an approach based on structure analysis for targeted mutagenesis of CE to alter its biocatalytic reaction to accumulate a desired product.

## Discussion

In this study, we applied structural analysis to enzyme active site pocket engineering for lactulose production. Specifically, end product stabilizing residues were replaced by amino acids that can inhibit product binding to increase production of lactulose, the isomeric byproduct of the epimerization reaction, at 65 °C, while the native epimeric product of the reaction, epilactose, was reduced. Generally, engineering active pocket residues is known to be difficult to change activity, because these residues play important roles in the reaction process. Likewise, in our case, combinations of Y114E along with active site residue N184, identified as another possible candidate in our structural analysis, did not show desirable results (Additional file 1: Table S3).

The replacement of Y114 by glutamate appears to involve an interesting environmental change whereby inhibiting the interaction between Y114 and the hydroxyl group in the C2 position of the mannose moiety prevents an epimerization of *cis*-enediol intermediate [12, 19, 20, 23]. The fact that prevention from interacting with the end product is necessary but not sufficient for lactulose production suggests the involvement of external environment conditions, such as high substrate concentration and high temperature that help to induce unstable epimerization or epilactose conversion by accelerating the reaction rate. Indeed, screening of heat resistant enzymes for lactulose production has been pursued by many researchers, including cell- or enzyme-immobilization reactors which are applicable for industrial use [4, 14, 15, 26–28].

Recently, Fujiwara et al. proposed epimerization mechanism in RmCE. In this proposed mechanism, His390 (corresponding to His377 in CsCE) is served as an acid/base catalyst for ring opening/closure by donating a proton to the O5 atom of the reducing end moiety of the substrate and abstracting the proton from the O1 atom of the same moiety, respectively [20]. In the direction of Glc to Man, initially deprotonated His390 of RmCE abstracts the proton from the O1 atom of the reducing end d-glucose residue, and then the resulting O5 anion accepts a proton from the protonated His390, in agreement with the following role of His390 as the base catalyst to form the *cis*-enediol intermediate. The mechanism of ring closure can be interpreted as the inverse reaction of ring opening. After the protonation/reprotonation step, rotation about the C2–C3 bond may occur again and position the O1 atom of the reducing end moiety of the substrate close to the O5 atom of the same moiety. Then, the electron pair on the O5 anion caused by deprotonation with His390 attacks the electron-deficient C1 atom to remake the C1–O5 bond [20]. Based on the proposed mechanism by Fujisawa, as an increase of pH by shifting temperature may affect to the role of His390 as acid/base, epimerization of lactose can be reduced by incubation at high temperature. Additionally, it is quite reasonable that the function of His390 acting on *cis*-enediol intermediate may be changed by replacing residues near active pocket. Taken together, it can be a possible explanation that His247 of CsCE, corresponding to His259 of RmCE, may transfer H2 to C1 rather than C2 of the *cis*-enediol intermediate by subtle environmental change of Y114E substitution, leading to an increase of isomerization/epimerization ratio under our incubation condition at high temperature [25].

CsCE, used as parent enzyme in this study, is known to convert aldose substrates with hydroxyl groups in the right-hand configuration at the C2 position to their epimers with the C2 hydroxyl groups in the left-hand configuration, while retaining left-hand configuration at the C3 position during epimerization [12]. Additionally, CsCE is known to have isomerization activity for both aldoses monosaccharides and cellobiose with up-shifting reaction parameters, i.e. enzyme amounts, incubation time and temperature, while CE from *E. cellulosolvens*, *R. albus*, and *B. fragilis* had no activity for monosaccharides and exhibited much higher epimerization activity for cellobiose than isomerization [18, 29, 30]. While not investigated in this study, we presume that RaCE may have a more rigid structure than CsCE, because RaCE is more thermostable than CsCE [19, 21]. This would explain how thermostable RaCE exhibits more specific epimerization activity than CsCE. Even though the relationship between thermostability and epimerization activity is not clearly explained, CsCE could be a much better candidate for this engineering compared to RaCE for this reason.

From a technological standpoint, a notable outcome of the studies described here was support of a hypothesis derived from structural analysis by engineering targeted active site residues for enhancing lactulose production. We demonstrated that interaction of the enzyme with product was inhibited by replacing Y114 with glutamate, leading to the release of lactulose. In particular, we showed increased lactulose production from lactose in Y114E compared to the parental enzyme at high temperature (65 °C) and high substrate concentration, while Y114E seemed to almost lose both epimerization and isomerization activity at 37 °C. Notably, the engineering described here has the potential to improve the enzyme activity and enlarge its industrial application.

## Additional file

**Additional file 1. Figure S1.** Three-dimentional structure of CsCE (PDB ID: 4Z4L). **Table S1.** Data and refinement statistics. **Table S2.** Comparison of isomerase/epimerase activities on CsCE N184X mutants at 37 °C and 65 °C. **Table S3.** Comparison of isomerase/epimerase activities on N184Q, N184D and N184R mutants combined with Y114E at 65 °C.

## Abbreviations

CE: cellobiose 2-epimerases
CsCE: *Caldicellulosiruptor saccharolyticus* CE
RaCE: *Ruminococcus albus* CE
RmCE: *Rhodothermus marinus* CE
AGE: *N*-acetylglucosamine 2-epimerase
