# Supplementary material for: Rational modification of substrate binding site by structure-based engineering of a cellobiose 2-epimerase in Caldicellulosiruptor saccharolyticus
Source: Microb Cell Fact. 2017 Dec 12;16:224. doi: 10.1186/s12934-017-0841-3 (PMC5726027; doi:10.1186/s12934-017-0841-3)
Supplement: Supplementary file 1 — Additional file 1. Figure S1. Three-dimentional structure of CsCE (PDB ID: 4Z4L). Table S1. Data and refinement statistics. Table S2. Comparison of isomerase/epimerase activities on CsCE N184X mutants at 37 °C and 65 °C. Table S3. Comparison of isomerase/epimerase activities on N184Q, N184D and N184R mutants combined with Y114E at 65 °C. [file 12934_2017_841_MOESM1_ESM.pdf]

Additional File 1

Rational modification of substrate binding site by structure-based engineering of a cellobiose 2-epimerase in *Caldicellulosiruptor saccharolyticus*

Ah-Reum Park, Jin-Sook Kim, Seung-Won Jang, Young-Gyun Park, Bong-Seong Koo, and Hyeon-Cheol Lee*

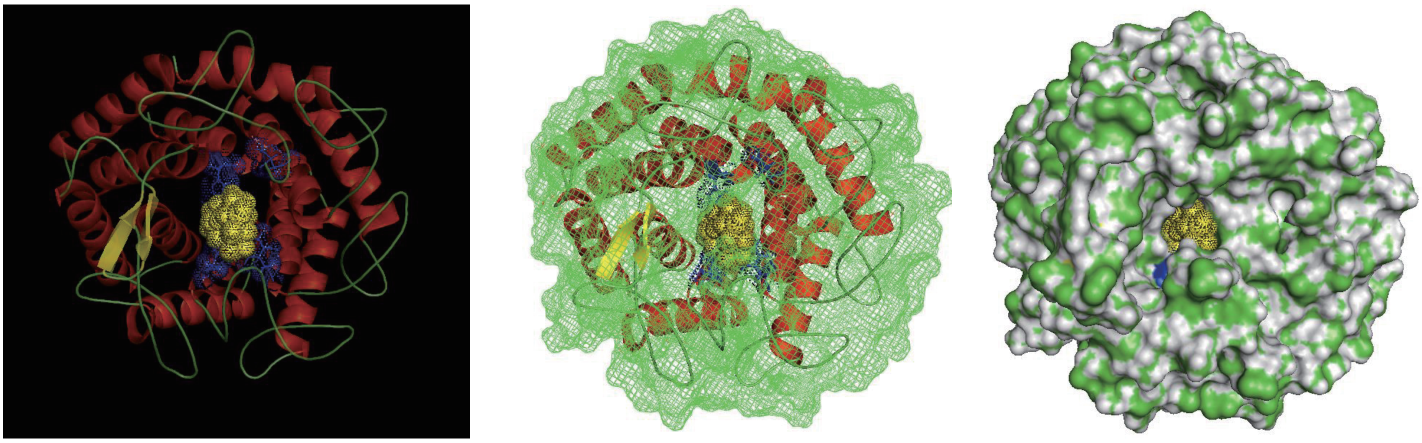

Figure S1. Three-dimentional structure of CsCE (PDB ID: 4Z4L). A: Cartoon model, B: Meshed filling model, C: surface filling model. This structure were displayed by using tools in PyMOL software. CsCE with lactulose was also optimized and refined by the same software. Helices, sheets and loops are designated in red, yellow and green, respectively. Whilte surface and green surface indicate hydrophilic region and hydrophobic region, respectively

Table S1. Data and refinement statistics.

**Property**	**Value**
Space group	P 21 21 21
Cell constants	a=55.8 Å b=75.5 Å c=91.4 Å<br>α=90.0° β=90.0° γ=90.0°
Resolution	30.0-1.67 Å
%Data completeness in resolution range (%)	83.1
R_{merge}	0.05
<I/σ(I)>	1.47 (at 1.67Å)
Refinement program	PHENIX
R, R_{free}	0.163, 0.198
RMSZ	Bond length 0.41<br>Bond angle 0.81
Ramachandran	Favored 92.7%<br>Allowed 7.0%<br>Outliers 0.3%
Total number of atoms (residue)	3432 (392)
Mean B factor(Å^2)	28.0

Table S2. Comparison of isomerase/epimerase activities on CsCE N184X mutants at 37°C and 65°C.

Amino acid substituted in N184	37°C		65°C	
	Isomerase activity (mM/min·µg)	Epimerase activity (mM/min·µg)	Isomerase activity (mM/min·µg)	Epimerase activity (mM/min·µg)
N (parent)	15.6±1.2	138±2.3	187±4.2	37.0±3.6
V	3.88±0.72	n.d	3.90±0.97	n.d
Y	1.87±0.33	n.d	1.85±0.12	n.d
S	n.d	n.d	n.d	n.d
A	n.d	n.d	n.d	n.d
K	n.d	n.d	n.d	n.d
G	1.95±0.11	n.d	1.91±0.33	n.d
D	3.88±0.99	n.d	9.72±0.17	n.d
C	n.d	n.d	n.d	n.d
L	1.92±0.82	n.d	1.92±0.22	n.d
E	3.91±0.98	n.d	15.6±1.39	n.d
P	1.93±0.33	n.d	1.85±0.43	n.d
I	n.d	n.d	n.d	n.d
W	n.d	n.d	n.d	n.d
T	n.d	n.d	n.d	n.d
R	n.d	n.d	n.d	n.d
F	1.94±0.12	9.7±1.2	1.91±0.12	9.7±1.2
M	3.90±0.37	n.d	3.91±0.67	n.d
Q	1.90±0.11	n.d	11.7±2.2	n.d
H	n.d	n.d	n.d	n.d

* Isomerase and epimerase activities were measured by quantifying lactulose and epilactose which were converted from 20 g/l lactose for 30 min at testing temperature.

** Values are means ± s.d. measured from three experimental replicates.

*** n.d not detected

Table S3. Comparison of isomerase/epimerase activities on N184Q, N184D and N184R mutants combined with Y114E at 65°C.

Mutant enzyme	65°C	
	Isomerase activity (mM/min·μg)	Epimerase activity (mM/min·μg)
Y114E	208±3.9	11.7±2.2
Y114E, N184Q	11.7±2.7	n.d
Y114E, N184D	25.3±3.5	n.d
Y114E, N184E	27.3±3.6	n.d

* Isomerase and epimerase activities were measured by quantifying lactulose and epilactose which were converted from 20 g/l lactose for 30 min at 65°C.

** Values are means ± s.d. measured from three experimental replicates.

*** n.d not detected
